# Personal

**Published:** 1901-05

**Authors:** 


### PERSONAL.

Dr. Abraham Jacobi, of New York, celebrated the 50th anniversary of his professional life at the New York Academy, April 5, 1901. He received and entertained a large number of his friends, physicians and laymen from 9 to 12 p.m., when he read to them a paper entitled, "German Text-books Half a Century Ago; History and Reminiscences."

It told of his experiences in the medical schools of the Universities of Greifswald, Göttingen and Bonn, teeming with anecdotes and recollections of his teachers and school friends.

The principal text-books which he had studied at the universities were arranged on the table in front of the chair usually occupied by the president of the academy. These book form an interesting collection, though not many in number. They contain on many pages annotations that were written by him. Dr. Jacobi gave these books to the library of the academy at the conclusion of his remarks, and altogether has made gifts of books to the library to the number of more than 1,000 volumes.

---

Dr. Edward B. Angell, of 295 Alexander Street, Rochester, N.Y., will sail for Europe Saturday, May 4, 1901, going direct to Naples. From there he will go to Vienna, thence to Berlin, afterward to Paris, and finally to London. These are his objective points, but other places will be visited either for study or pleasure. Dr. Angell will return in September and will take part in the meeting of the Medical Association of Central New York, to be held at Buffalo in October.

---

Dr. Richard Curran, of Rochester, N.Y., has been visiting the physicians of Buffalo during the past month, in the interest of the Duffy Malt Whiskey Co. Dr. Curran was mayor of Rochester for a term and also served in the legislature. He served as a surgeon of cavalry in the civil war and won a medal of honor.

---

Dr. George Blumer, of the Bender Laboratory, Albany, has been appointed director of the new bureau of bacteriology and pathology in the newly created state department of health. It will be one of the most important bureaus under the health commissioner, as tests will be made to detect tuberculosis, diphtheria and other diseases.

---

Dr. William Warren Potter, of Buffalo, has accepted the invitation of the faculty of the Hospital College of Medicine, of Louisville, Ky., to deliver the doctorate address at the commencement exercises of the college to be held at Macauley's Theatre, the last week in June, 1901.

Dr. Charles L. Scudder, of Boston, surgeon of the Massachusetts General Hospital, addressed the meeting of the Buffalo Academy of Medicine in the Buffalo Library Building, Tuesday evening, April 2, 1901, on "Diagnosis and Treatment of Contusions of the Abdomen." It was an able presentation of a most important subject. Dr. Scudder was entertained at dinner before the meeting by Dr. Chauncey P. Smith, who invited a number of medical friends to meet the guest of honor.

---

Dr. John H. Grant, of Buffalo, commissioner of agriculture for the State of New York, has removed his residence from 176 Virginia Street, Buffalo, to Union Street, Hamburg. His Buffalo address and office are at No. 893 Ellicott Square, Buffalo.

---

Dr. and Mrs. G. A. Himmelsbach, of Buffalo, will sail in June for a three months' trip to Europe. They will travel extensively on the continent and Dr. Himmelsbach will visit some of the clinics in Vienna and other medical centers.

---

Dr. Francis M. O'Gorman, of Buffalo, has removed from the Carmichael to his new office and residence, No. 1298 Jefferson St., (formerly Dr. C. B. LeVan's.) Hours: 8 to 9.30 a. m; 1 to 3, 6 to 8 p.m; Telephone.

---

Dr. R. A. Patchin, U. B., '69, a prominent physician of Des Moines, Ia., visited Buffalo recently, calling on Dr. Conrad Diehl and Dr. George W. Pattison, of Chippewa Street, classmates.

